# The non-high-density lipoprotein cholesterol to high-density lipoprotein cholesterol ratio is associated with early onset type 2 diabetes with NAFLD: a cross-sectional study in China

**DOI:** 10.3389/fnut.2025.1662125

**Published:** 2025-10-31

**Authors:** Nini Wu, Yingqun Ni, Rilong Huang, Junhua Wang, Meimei Li, Haiying Hu, Yuanyuan Zhang, Juyi Li, Jinjun Zhang

**Affiliations:** ^1^Department of Endocrinology, Geriatrics Center, The First Affiliated Hospital of Anhui University of Chinese Medicine, Hefei, Anhui, China; ^2^The First Clinical Medical College, Anhui University of Chinese Medicine, Hefei, Anhui, China; ^3^Department of Endocrinology, The First Affiliated Hospital of Anhui University of Chinese Medicine, Hefei, Anhui, China; ^4^Health Management Center, The First Affiliated Hospital of Anhui University of Chinese Medicine, Hefei, Anhui, China

**Keywords:** NHHR, early onset, type 2 diabetes mellitus, NAFLD, cross-sectional study

## Abstract

**Background:**

The non-high-density lipoprotein cholesterol to high-density lipoprotein cholesterol ratio (NHHR) is a novel composite lipid biomarker associated with metabolic diseases. This study aims to explore the potential relationship between early-onset type 2 diabetes mellitus combined with NAFLD and NHHR.

**Methods:**

A total of 1,158 patients with type 2 diabetes were enrolled and categorized by age at onset into the early-onset type 2 diabetes (EOT2D) group (260 cases, <40 years) and the late-onset type 2 diabetes (LOT2D) group (898 cases, ≥40 years). The EOT2D group was further subdivided into those with NAFLD (136 cases) and those without NAFLD (124 cases). Multivariate logistic regression assessed the association between NHHR and EOT2D, as well as EOT2D combined with NAFLD. Additionally, we employed subgroup analysis, interaction analysis, smoothing curve fitting, and receiver operating characteristic curve analysis to further explore risk factors for EOT2D and EOT2D combined with NAFLD.

**Results:**

This cross-sectional study included 260 EOT2D patients and 898 LOT2D patients. After adjusting for confounding factors, the study found that for every 1-unit increase in NHHR, the risk of EOT2D increased by 22% (OR = 1.22, 95% CI: 1.05–1.42), and the risk of EOT2D with NAFLD increased by 41% (OR = 1.41, 95% CI: 1.01–1.98). The AUC for diagnosing EOT2D with NAFLD using NHHR was 0.658 (95% CI: 0.592–0.724), with an optimal cutoff of 3.105 (sensitivity 77.21%, specificity 50.00%).

**Conclusion:**

Our study suggests that NHHR is a risk factor for EOT2D and EOT2D combined with NAFLD, serving as a reference indicator for screening this high-risk population.

## Introduction

The prevalence of type 2 diabetes mellitus (T2DM) has been on the rise globally over the past few decades (1), which may be the cause of lethal events such as stroke, heart failure, kidney disease, and retinopathy (2). In recent years, the incidence of early-onset type 2 diabetes (EOT2D) has been increasing annually, especially after the COVID-19 pandemic, and this incidence has risen dramatically (3). Compared with late-onset type 2 diabetes (LOT2D), patients with early-onset type 2 diabetes (EOT2D) exhibit a more rapid decline in *β*-cell secretory function, faster disease progression, higher risk of complications, and earlier onset of complications (4, 5). Several studies have shown that nonalcoholic fatty liver disease (NAFLD) and T2DM are two frequently co-existing pathologic states and that there is a bidirectional relationship between them (6), the prevalence of NAFLD was significantly higher in T2DM patients than in non-T2DM patients (7). Researchers are now proposing metabolic dysfunction-associated fatty liver disease (MAFLD) as a more instructive medical diagnosis to describe this disease, which emphasizes the causative role of metabolic dysfunction in the development and progression of this highly prevalent disease. MAFLD emphasizes the role of metabolic dysfunction in the development and progression of this highly prevalent disease, and T2DM in combination with NAFLD is a case of MAFLD (8). In addition, NAFLD is more common in patients with EOT2D than LOT2D (9). Relevant studies have shown that the association between T2DM and increased risk of patient death is more pronounced in younger patients with MAFLD (10), and that patients with T2DM combined with NAFLD who ultimately develop nonalcoholic steatohepatitis (NASH) are also relatively younger (11), and that there is no approved of clearly effective drug therapy.

The strong association between dyslipidemia and T2DM and NAFLD (12, 13). The development of NAFLD is associated with severe abnormalities in hepatic lipid metabolism (14). Insulin resistance (IR) is central to the development of NAFLD and T2DM and the main physiopathological link between the two, triggering an increase in free fatty acids in peripheral adipose tissue and promoting dyslipidemia (15). Lipid disorders are strongly associated with insulin resistance and disorders of glucose metabolism (16, 17). Metabolic dyslipidemia is very common in patients with type 2 diabetes (40%) (18), Hypercholesterolemia and lowered high-density lipoprotein cholesterol (HDL-C) occur more often in patients with diabetes mellitus (19). The non-high-density lipoprotein cholesterol to high-density lipoprotein cholesterol ratio (NHHR) is a novel indicator of atherogenic lipids that has received much attention in recent years. It provides a more comprehensive assessment of lipid metabolism disorders (20), which is closely related to the correlation and predictive value of cardiovascular diseases, stroke, inflammatory diseases, etc. (21–23). In addition, studies have shown that NHHR is superior to traditional lipid markers in the diagnosis of insulin resistance (IR) and metabolic syndrome (Mts) (12, 24).

Although previous studies have suggested associations between NHHR and both T2DM and NAFLD, the predictive value of NHHR for the high-risk population with EOT2D complicated by NAFLD remains unclear. Given that patients with EOT2D and NAFLD typically exhibit more severe metabolic abnormalities and poorer clinical outcomes, early identification of this group holds significant clinical importance. Therefore, this study aims to investigate the association between NHHR and EOT2D, as well as their co-occurrence with NAFLD, with the goal of providing new evidence-based support for the early identification and intervention of this high-risk population.

## Materials and methods

### Study population

**Figure 1:**
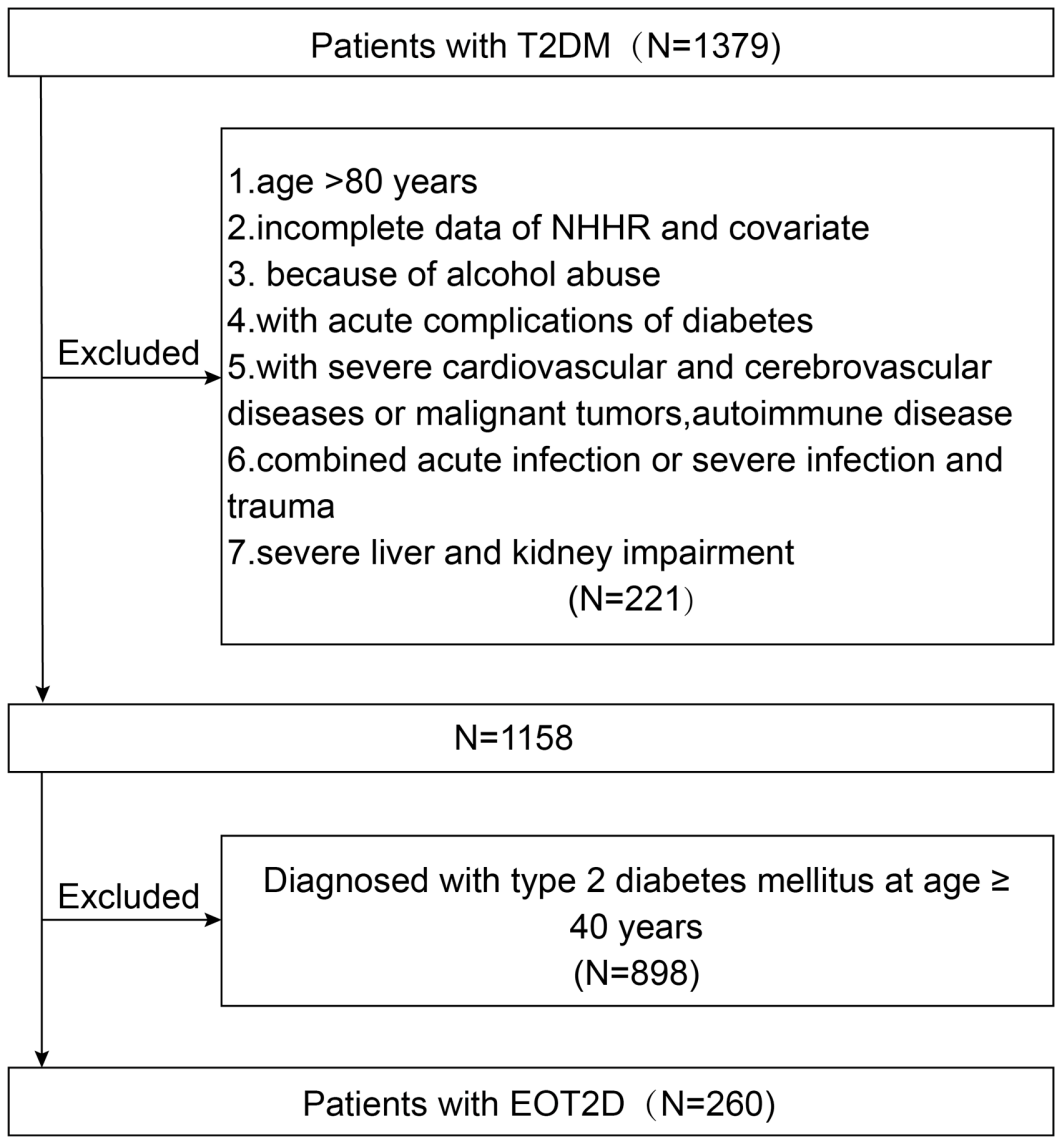
Flow chart of patient recruitment.

In this study, 1,158 patients with T2DM admitted to the Department of Endocrinology of the First Affiliated Hospital of Anhui University of Traditional Chinese Medicine from April 2023 to April 2025 were retrospectively clinically analyzed. Initially, 1,379 participants with T2DM were selected. The exclusion criteria were (1) age >80 years; (2) with acute complications of diabetes; (3) with severe cardiovascular or cerebrovascular diseases or malignant tumors, autoimmune diseases; (4) with acute or severe infections, trauma; (5) with severe hepatic or renal impairment; (6) with excessive alcohol consumption (defined as an average of >70 g per week for women, or >140 g per week for men) (25); (7) Missing TC or HDL-C data prevented the calculation of NHHR. One thousand one hundred fifty-eight participants were finally included in the study (Figure 1). This study was approved by the Medical Ethics Committee of the First Affiliated Hospital of Anhui University of Traditional Chinese Medicine (No. 2025AH-103) and followed the ethical guidelines of the Declaration of Helsinki (1964).

### NHHR calculation and assessment of early onset type 2 diabetes and NAFLD and subgroups

NHHR was calculated as non-HDL-C/HDL-C, an exposure variable, and was derived from the participants’ lipid values, with non-HDL-C being the serum TC value minus HDL-C. Lipids were measured by a Roche Cobas 6,000 automatic biochemical analyzer for numerical measurements. The diagnosis of T2DM was based on the diagnostic criteria proposed by the WHO Expert Committee on Diabetes Mellitus in 1999. Year of type 2 diabetes diagnosis age less than 40 years is defined as early onset type 2 diabetes (EOT2D) and 40 years or older is defined as late onset type 2 diabetes (LOT2D) (5). Diagnosis of NAFLD is based on an ultrasound examination of the upper abdomen, which is done using a Myers Nuewa R9 Pro color ultrasound diagnostic machine (abdominal probe, probe frequency 3.5–5.0 MHz), and the diagnosis of fatty liver is confirmed by a combination of two experienced ultrasonographers. The diagnosis of fatty liver by ultrasound is based on at least two of the following three abnormalities: diffuse hepatic echogenic enhancement (“bright”)—liver echogenicity is greater than that of the kidneys or spleen; vascular blurring; and deep attenuation of the ultrasound signal (26). Two groups were categorized according to whether the age of onset of diabetes was less than 40 years, one group of EOT2D patients (260 cases) and one group of LOT2D patients (898 cases). The EOT2D group was further divided into the group with NAFLD (136 cases) and the group without NAFLD (124 cases) based on the results of an ultrasound examination of the upper abdomen. Subgroup analyses categorized individuals by age (≤45, 45–60, >60), sex (male, female), smoking status (yes, no), BMI (<24, ≥24), and history of hypertension (yes, no).

### General clinical information and laboratory tests

To investigate the independent relationship between NHHR and the risk of EOT2D comorbid NAFLD, we recorded some basic clinical information such as age, gender, duration of diabetes, history of smoking, history of hypertension, presence or absence of comorbid diabetic kidney disease (DKD), and body mass index (BMI), which was calculated as the square of the body weight (kg) divided by the height (m). Laboratory parameters included fasting insulin (FINS, uIU/mL), fasting C-peptide (FCP, μg/L), fasting glucose (FBG, mmol/L), glycosylated hemoglobin (HbA1c, %), alanine aminotransferase (ALT, U/L), aspartate aminotransferase (AST, U/L), albumin (ALB, g/L), gamma-glutamyl transferase (GGT, U/L), urea nitrogen (BUN, mmol/L), creatinine (Cr, μmol/L), uric acid (UA, μmol/L), triglycerides (TG, mmol/L), total cholesterol (TC, mmol/L), HDL-C, mmol/L, LDL-C, mmol/L, and blood pressure (BP/mmHg), white blood cell count (WBC, 10_9_/L), neutrophil count (N, 10_9_/L), lymphocyte count (L, 10_9_/L), monocyte count (M, 10_9_/L), platelet count (PLT, 10_9_/L), and urine albumin creatinine ratio (UACR, mg/g). Smoking status was determined based on lifetime smoking of at least 100 cigarettes. History of hypertension was defined as a self-reported physician diagnosis or blood pressure over 140/90 mmHg.

### Statistical analysis

Normally distributed continuous variables are expressed as mean ± standard deviation, non-normally distributed data are expressed as median (interquartile spacing), and comparisons between 2 groups were performed using the independent samples *t*-test or Mann–Whitney *U*-test, and comparisons between multiple groups were performed using the Kruskal–Wallis H rank-sum test. Categorical variables were expressed as counts and percentages (%), and differences between groups were tested using the chi-square test. Three different logistic regression models were used to analyze the relationship between NHHR and EOT2D and between NHHR and EOT2D combined with NAFLD, and the odds ratios (OR) and their 95% confidence intervals (CI) were calculated and adjusted for a range of covariates for the three models. In addition, trend tests, subgroup analyses, and interaction analyses were performed. Smoothed curves were used to explore the associations between NHHR and EOT2D and EOT2D combined with NAFLD, and the validity of NHHR in assessing the risk of developing EOT2D combined with NAFLD was analyzed using subject work characteristics (ROC) curves. To account for multiple comparisons and determine statistical significance, the Bonferroni correction was applied, setting the adjusted significance threshold at *α* = 0.01 for subgroup interaction tests (calculated as 0.05 divided by five subgroups). All statistical calculations were performed using R Statistical Software version 4.2.0 in conjunction with Empower software (www.empowerstats.com), and statistical significance was set at *P* < 0.05.

## Results

### Participants characteristics

Of the 1,158 participants with T2DM, 260 were in the EOT2D group and 898 in the LOT2D group. 73.46% (191/260) of the EOT2D group and 56.79% (510/898) of the LOT2D group were male. Compared with the patients in the LOT2D group, the duration of diabetes, NHHR, the proportion of smokers, the prevalence of diabetic nephropathy, BMI, FBG, HbA1c, ALT, ALB, GGT, UA, TG, WBC, N, and PLT levels were significantly higher in the EOT2D group (*P* < 0.05), and HDL-C was lower than in the LOT2D group (*P* < 0.05) (Table 1). The levels of FINS, AST, BUN, Cr, TC, LDL-C, and UACR were not significantly different between the two groups of participants. Supplementary Table S1 describes the baseline characteristics of the subjects based on NHHR quartiles, and significant differences were observed between NHHR quartiles and baseline characteristics. Individuals in the higher quartile group had a higher prevalence of NAFLD and EOT2D compared with the Q1 group.

**Table 1:** Baseline clinical characteristics according to EOT2D status.

Variable	Total	EOT2D	LOT2D	*P*-value
	*N* = 1,158	*N* = 260	*N* = 898	
Age (years)	59.29 (11.28)	47.89 (11.27)	62.59 (8.87)	<0.001
Sex (%)				<0.001
Male	701 (60.54)	191 (73.46)	510 (56.79)	
Female	457 (39.46)	69 (26.54)	388 (43.21)	
Smoking (%)				0.003
Yes	255 (22.02)	75 (28.85)	180 (20.04)	
No	903 (77.98)	185 (71.15)	718 (79.96)	
Hypertension (%)				<0.001
Yes	650 (56.13)	116 (44.62)	534 (59.47)	
No	508 (43.87)	144 (55.38)	364 (40.53)	
Diabetic kidney disease (%)				0.045
Yes	305 (26.34)	81 (31.15)	224 (24.94)	
No	853 (73.66)	179 (68.85)	674 (75.06)	
Disease duration (months)	120.00 (36.00–180.00)	120.00 (48.00–219.00)	108.00 (36.00–168.00)	<0.001
BMI (kg/m^2^)	25.05 ± 3.45	25.77 ± 3.45	24.84 ± 3.42	<0.001
FINS (uIU/mL)	8.28 (5.44–12.97)	8.33 (5.37–13.85)	8.25 (5.46–12.82)	0.296
FCP (μg/L)	1.97 (1.33–2.84)	1.88 (1.14–2.75)	1.99 (1.38–2.85)	0.054
FBG (mmol/L)	7.89 ± 2.85	8.20 ± 2.95	7.80 ± 2.82	0.045
HbA1c (%)	8.11 ± 1.79	8.35 ± 1.74	8.04 ± 1.80	0.015
ALT (U/L)	17.80 (13.30–25.50)	19.80 (14.07–28.90)	17.30 (13.20–24.65)	0.007
AST (U/L)	20.63 ± 7.64	20.19 ± 7.80	20.76 ± 7.59	0.293
ALB (g/L)	40.28 ± 3.59	40.89 ± 3.68	40.10 ± 3.55	0.002
GGT (U/L)	22.00 (16.00–33.00)	24.50 (16.00–37.00)	21.00 (15.00–32.00)	0.008
BUN (mmol/L)	5.89 ± 1.63	5.79 ± 1.54	5.92 ± 1.66	0.268
Cr (μmol/L)	66.15 ± 17.87	65.96 ± 17.70	66.20 ± 17.93	0.850
UA (μmol/L)	323.37 ± 86.99	334.67 ± 80.13	320.10 ± 88.66	0.017
TG (mmol/L)	1.56 (1.12–2.37)	1.70 (1.21–2.64)	1.52 (1.08–2.29)	<0.001
TC (mmol/L)	4.76 ± 1.15	4.83 ± 1.13	4.74 ± 1.15	0.245
HDL-C (mmol/L)	1.18 ± 0.28	1.11 ± 0.25	1.19 ± 0.28	<0.001
LDL-C (mmol/L)	2.98 ± 0.79	3.06 ± 0.78	2.95 ± 0.79	0.058
NHHR	3.15 ± 1.05	3.45 ± 1.06	3.07 ± 1.03	<0.001
WBC (10^9^/L)	6.12 ± 1.48	6.28 ± 1.51	6.08 ± 1.47	0.033
N (10^9^/L)	3.46 ± 1.11	3.54 ± 1.08	3.43 ± 1.11	0.044
L (10^9^/L)	2.00 ± 0.62	2.07 ± 0.64	1.99 ± 0.62	0.067
M (10^9^/L)	0.46 ± 0.15	0.47 ± 0.15	0.46 ± 0.15	0.380
PLT (10^9^/L)	197.37 ± 49.37	207.54 ± 51.83	194.42 ± 48.26	<0.001
UACR (mg/g)	11.08 (5.62–33.12)	11.71 (5.52–39.23)	10.91 (5.63–29.70)	0.538

BMI, body mass index; FINS, fasting insulin; FCP, fasting C-peptide; FBG, fasting blood glucose; HbA1c, Hemoglobin A1c; ALT, alanine transaminase; AST, aspartate transaminase; ALB, albumin; GGT, gamma-glutamyl transferase; BUN, blood urea nitrogen; Cr, serum creatinine; UA, serum uric acid; TG, triglyceride; TC, total cholesterol; HDL-C, high density lipoprotein cholesterol; LDL-C, low density lipoprotein cholesterol; NHHR, non-high-density lipoprotein cholesterol to high-density lipoprotein cholesterol ratio; WBC, white Blood Cell Differential; N, neutrophil count; L, lymphocyte count; M, monocyte count; PLT, platelet count; UACR, urine albumin-to-creatinine ratio.

### The association between NHHR with EOT2D

**Figure 2:**
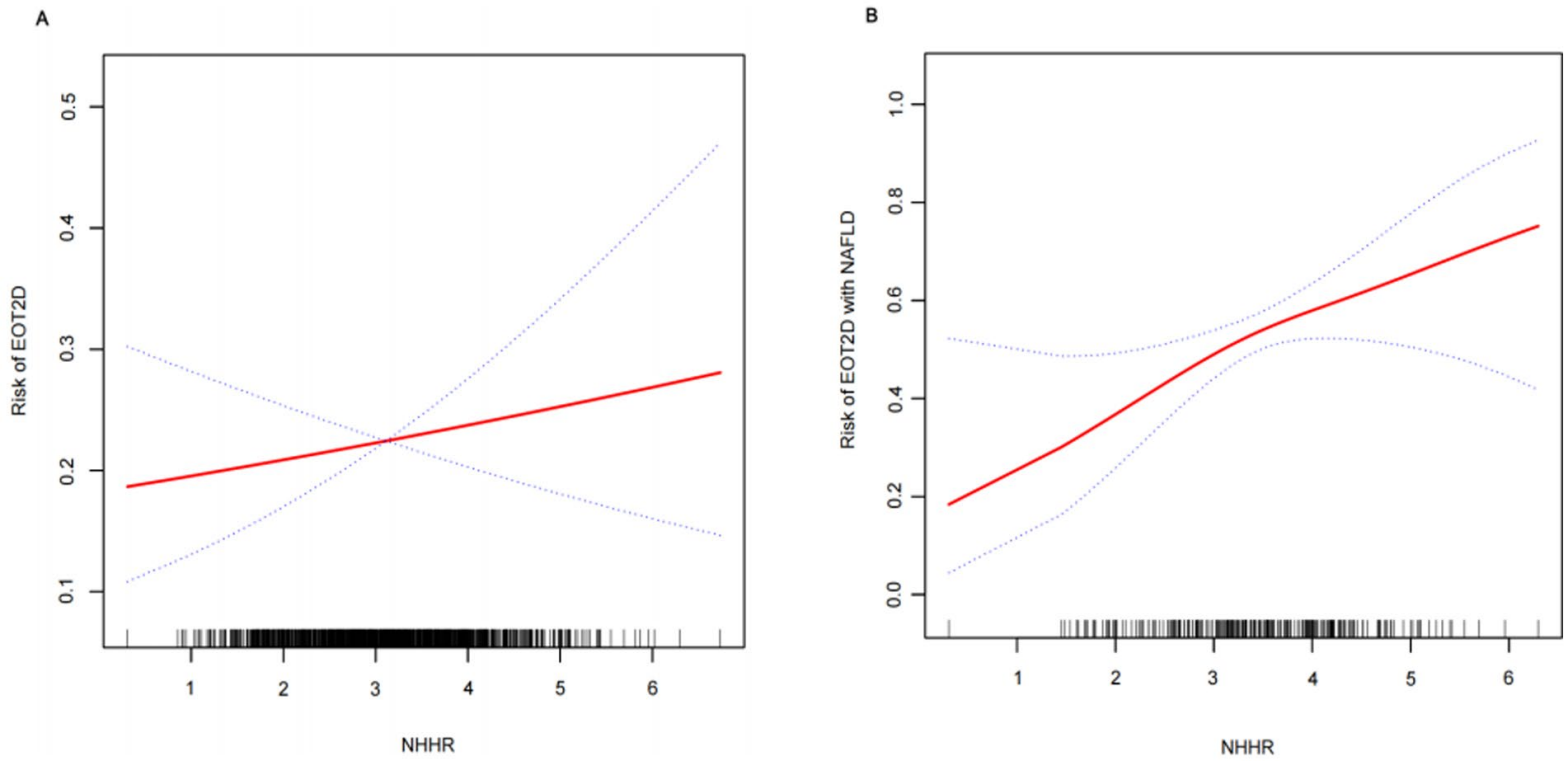
Smooth curve fitting depicting the association between NHHR and EOT2D, as well as EOT2D complicated by NAFLD.

Multifactorial logistic regression modeling confirmed that NHHR was an independent risk factor for EOT2D, and the results of the analysis are presented in Table 2. The results showed that in model 1, which was not adjusted for any covariates, there was a 39% increase in the risk of EOT2D for each 1-unit increase in NHHR (OR = 1.39; 95% CI, 1.22–1.59; *P* < 0.0001). In model 2, which was adjusted for sex, duration of diabetes, smoking status, BMI, and history of hypertension, the risk increased by 30% (OR = 1.30; 95% CI, 1.13–1.49; *P* = 0.0002). Model 3 was further adjusted for HbA1c, ALT, AST, UA, WBC, PLT, and UACR, and the risk of EOT2D increased by 21%, suggesting that NHHR was independently associated with EOT2D. Analyzing NHHR as a categorical variable (quartiles), participants in the highest quartile (Q4) had a significantly higher risk of developing EOT2D than those in the lowest quartile (Q1), with a 1.17-fold increase in risk in model 3 (OR = 2.17; 95% CI, 1.36–3.44; *P* = 0.0010). In addition, trend analysis revealed that the risk of EOT2D increased progressively with increasing NHHR quartiles (*P* for trend = 0.0011). In addition, smoothed curve fitting showed a linear correlation between NHHR and EOT2D (*P* = 0.432), with no significant threshold or saturation effects (Figure 2A).

**Table 2:** Relationship between NHHR and early-onset diabetes mellitus in different models.

	Model 1		Model 2		Model 3	
	OR (95% CI)	*P*-value	OR (95% CI)	*P*-value	OR (95% CI)	*P*-value
NHHR	1.39 (1.22, 1.59)	<0.0001	1.30 (1.13, 1.49)	0.0002	1.21 (1.04, 1.40)	0.0119
Q1	Ref	Ref	Ref	Ref	Ref	Ref
Q2	1.47 (0.94, 2.29)	0.0900	1.49 (0.94, 2.36)	0.0896	1.33 (0.83, 2.12)	0.2379
Q3	1.89 (1.23, 2.90)	0.0039	1.74 (1.11, 2.72)	0.0158	1.41 (0.89, 2.25)	0.1432
Q4	3.19 (2.11, 4.82)	<0.0001	2.86 (1.85, 4.42)	<0.0001	2.17 (1.36, 3.44)	0.0010
*P* for trend		<0.0001		<0.0001		0.0011

Model 1: Non-adjusted.

Model 2: Adjusted for sex, diabetes duration, smoking, BMI, hypertension.

Model 3: Adjusted for sex, diabetes duration, smoking, BMI, hypertension, HbA1c, ALT, AST, UA, WBC, PLT, UACR.

CI, confidence interval; Ref, reference.

### Baseline characteristics of EOT2D patients by NAFLD status

Patients with EOT2D were categorized into two groups according to the presence or absence of comorbid NAFLD and the baseline characteristics of the participants were compared. There were 124 patients in the EOT2D uncomplicated NAFLD group, 71.77% of males and 28.23% of females, who had NHHR values of 3.14 ± 0.91. There were 136 patients in the EOT2D complicated NAFLD group, 75% of males and 25% of females, who had an NHHR value was 3.74 ± 1.11. Compared with the patients in the EOT2D-uncombined NAFLD group, the EOT2D-combined NAFLD group had significantly higher levels of BMI, FINS, FCP, FBG, HbA1c, ALT, AST, ALB, GGT, UA, TG, TC, NHHR, WBC, N, L, M, and PLT (*P* < 0.05) (see Table 3). Supplementary Table S2 describes the baseline characteristics of EOT2D patients based on NHHR quartiles, and significant differences in NHHR quartiles and baseline characteristics were observed. The prevalence of NAFLD was higher in the higher quartile group of EOT2D patients compared with the Q1 group (see Table 3).

**Table 3:** Baseline characteristics of early-onset T2DM patients stratified by NAFLD status.

Variable	Total	EOT2D without NAFLD	EOT2D with NAFLD	*P*-value
	*N* = 260	*N* = 124	*N* = 136	
Age (years)	47.89 (11.27)	49.16 (10.63)	46.73 (11.74)	0.082
Sex (%)				0.556
Male	191 (73.46)	89 (71.77)	102 (75.00)	
Female	69 (26.54)	35 (28.23)	34 (25.00)	
Smoking (%)				0.833
Yes	75 (28.85)	35 (28.23)	40 (29.41)	
No	185 (71.15)	89 (71.77)	96 (70.59)	
Hypertension (%)				0.504
Yes	116 (44.62)	58 (46.77)	58 (42.65)	
No	144 (55.38)	66 (53.23)	78 (57.35)	
Diabetic kidney disease (%)				0.330
Yes	81 (31.15)	35 (28.23)	46 (33.82)	
No	179 (68.85)	89 (71.77)	90 (66.18)	
Disease duration (months)	120.00 (48.00–219.00)	120.00 (36.00–216.00)	120.00 (48.00–228.00)	0.682
BMI (kg/m^2^)	25.77 ± 3.45	24.65 ± 3.08	26.80 ± 3.46	<0.001
FINS (uIU/mL)	8.33 (5.37–13.85)	6.29 (4.21–10.08)	11.10 (7.06–17.00)	<0.001
FCP (μg/L)	1.88 (1.14–2.75)	1.60 (0.87–2.18)	2.21 (1.62–3.31)	<0.001
FBG (mmol/L)	8.20 ± 2.95	7.51 ± 2.61	8.82 ± 3.10	<0.001
HbA1c (%)	8.35 ± 1.74	8.08 ± 1.84	8.59 ± 1.62	0.017
ALT (U/L)	19.80 (14.07–28.90)	16.50 (13.02–22.38)	22.80 (16.60–36.42)	<0.001
AST (U/L)	20.19 ± 7.80	18.52 ± 6.54	21.71 ± 8.54	<0.001
ALB (g/L)	40.89 ± 3.68	39.93 ± 3.32	41.77 ± 3.77	<0.001
GGT (U/L)	24.50 (16.00–37.00)	19.00 (14.00–29.00)	29.00 (20.00–46.25)	<0.001
BUN (mmol/L)	5.79 ± 1.54	5.93 ± 1.51	5.67 ± 1.55	0.170
Cr (μmol/L)	65.96 ± 17.70	66.58 ± 19.61	65.39 ± 15.81	0.589
UA (μmol/L)	334.67 ± 80.13	315.27 ± 72.75	352.35 ± 82.68	<0.001
TG (mmol/L)	1.70 (1.21–2.64)	1.42 (1.12–2.26)	2.08 (1.51–3.19)	0.002
TC (mmol/L)	4.83 ± 1.13	4.69 ± 1.26	4.96 ± 0.98	0.030
HDL-C (mmol/L)	1.11 ± 0.25	1.15 ± 0.27	1.07 ± 0.22	0.050
LDL-C (mmol/L)	3.06 ± 0.78	2.99 ± 0.88	3.13 ± 0.67	0.147
NHHR	3.45 ± 1.06	3.14 ± 0.91	3.74 ± 1.11	<0.001
WBC (10^9^/L)	6.28 ± 1.51	6.01 ± 1.50	6.52 ± 1.49	0.006
N (10^9^/L)	3.54 ± 1.08	3.45 ± 1.11	3.63 ± 1.05	0.047
L (10^9^/L)	2.07 ± 0.64	1.94 ± 0.62	2.18 ± 0.64	0.002
M (10^9^/L)	0.47 ± 0.15	0.43 ± 0.15	0.50 ± 0.15	<0.001
PLT (10^9^/L)	207.54 ± 51.83	196.69 ± 49.47	217.43 ± 52.13	0.001
UACR (mg/g)	11.71 (5.52–39.23)	11.68 (4.85–36.06)	11.71 (6.37–41.54)	0.323

BMI, body mass index; FINS, fasting insulin; FCP, fasting C-peptide; FBG, fasting blood glucose; HbA1c, Hemoglobin A1c; ALT, alanine transaminase; AST, aspartate transaminase; ALB, albumin; GGT, gamma-glutamyl transferase; BUN, blood urea nitrogen; Cr, serum creatinine; UA, serum uric acid; TG, triglyceride; TC, total cholesterol; HDL-C, high density lipoprotein cholesterol; LDL-C, low density lipoprotein cholesterol; NHHR, non-high-density lipoprotein cholesterol to high-density lipoprotein cholesterol ratio; WBC, white Blood Cell Differential; N, neutrophil count; L, lymphocyte count; M, monocyte count; PLT, platelet count; UACR, urine albumin-to-creatinine ratio.

### Association between NHHR and EOT2D complicated by NAFLD

Multivariate logistic regression analysis was performed to investigate the correlation between NHHR and NAFLD combined with EOT2D. Table 4 shows a statistically significant positive correlation between NHHR and NAFLD combined with EOT2D across three models: the unadjusted Model 1 yielded an odds ratio (OR) of 1.90 (95% CI, 1.43–2.54); Model 2, adjusted for sex, diabetes duration, smoking status, BMI, and history of hypertension, yielded an OR of 1.81 (95% CI, 1.34–2.45); and Model 3, further adjusted for HbA1c, ALT, AST, UA, WBC, PLT, and UACR, yielded an OR of 1.48 (95% CI, 1.06–2.05). This positive association remained consistent across all models. To further explore the relationship between NHHR and NAFLD combined with EOT2D, we categorized NHHR into quartiles. In Model 2, higher NHHR levels in the Q3 and Q4 groups were significantly and positively associated with an increased risk of NAFLD combined with EOT2D. In Model 3, compared to the lowest quartile (Q1) of NHHR, the highest quartile (Q4) remained significantly associated with an increased risk of NAFLD combined with EOT2D (OR = 3.69; 95% CI: 1.38–9.85). Trend analysis revealed that the risk of developing NAFLD in EOT2D patients increased significantly with ascending NHHR quartiles (*P* for trend = 0.0236). Furthermore, smooth curve fitting indicated a linear relationship between NHHR and NAFLD combined with EOT2D (*P* = 0.131), with no obvious threshold or saturation effect observed (Figure 2B).

**Table 4:** Association between NHHR and EOT2D combined with NAFLD.

	Model 1		Model 2		Model 3	
	OR (95% CI)	*P*-value	OR (95% CI)	*P*-value	OR (95% CI)	*P*-value
NHHR	1.90 (1.43, 2.54)	<0.0001	1.81 (1.34, 2.45)	0.0001	1.48 (1.06, 2.05)	0.0198
Q1	Ref	Ref	Ref	Ref	Ref	Ref
Q2	2.16 (0.88, 5.27)	0.0921	1.93 (0.75, 4.94)	0.1713	1.80 (0.66, 4.87)	0.2492
Q3	4.73 (1.99, 11.27)	0.0005	4.44 (1.78, 11.08)	0.0014	3.69 (1.38, 9.85)	0.0092
Q4	5.17 (2.26, 11.79)	<0.0001	4.47 (1.88, 10.63)	<0.0007	2.75 (1.07, 7.07)	0.0350
*P* for trend		<0.0001		<0.0002		0.0236

Model 1: Non-adjusted.

Model 2: Adjusted for sex, diabetes duration, smoking, BMI, hypertension.

Model 3: Adjusted for sex, diabetes duration, smoking, BMI, hypertension, HbA1c, ALT, AST, UA, WBC, PLT, UACR.

CI, confidence interval; Ref, reference.

### Gender-stratified analysis

Multivariate logistic regression models confirmed that NHHR is an independent risk factor for EOT2D and EOT2D combined with NAFLD in the male subgroup, with results presented in Supplementary Table S3. Supplementary Table S3(1) indicates that in the male subgroup, each one-unit increase in NHHR is associated with a 32% higher risk of EOT2D in Model 1. In Model 2, the risk increased by 30%; in Model 3, the risk increased by 21%, indicating that NHHR is independently associated with EOT2D in the male subgroup. Supplementary Table S3(2) shows a statistically significant positive correlation between NHHR and EOT2D with NAFLD in all three models within the male subgroup. In contrast, the results in Supplementary Table S4 indicate that this association is not statistically significant in the female subgroup.

### Subgroup analysis

**Figure 3:**
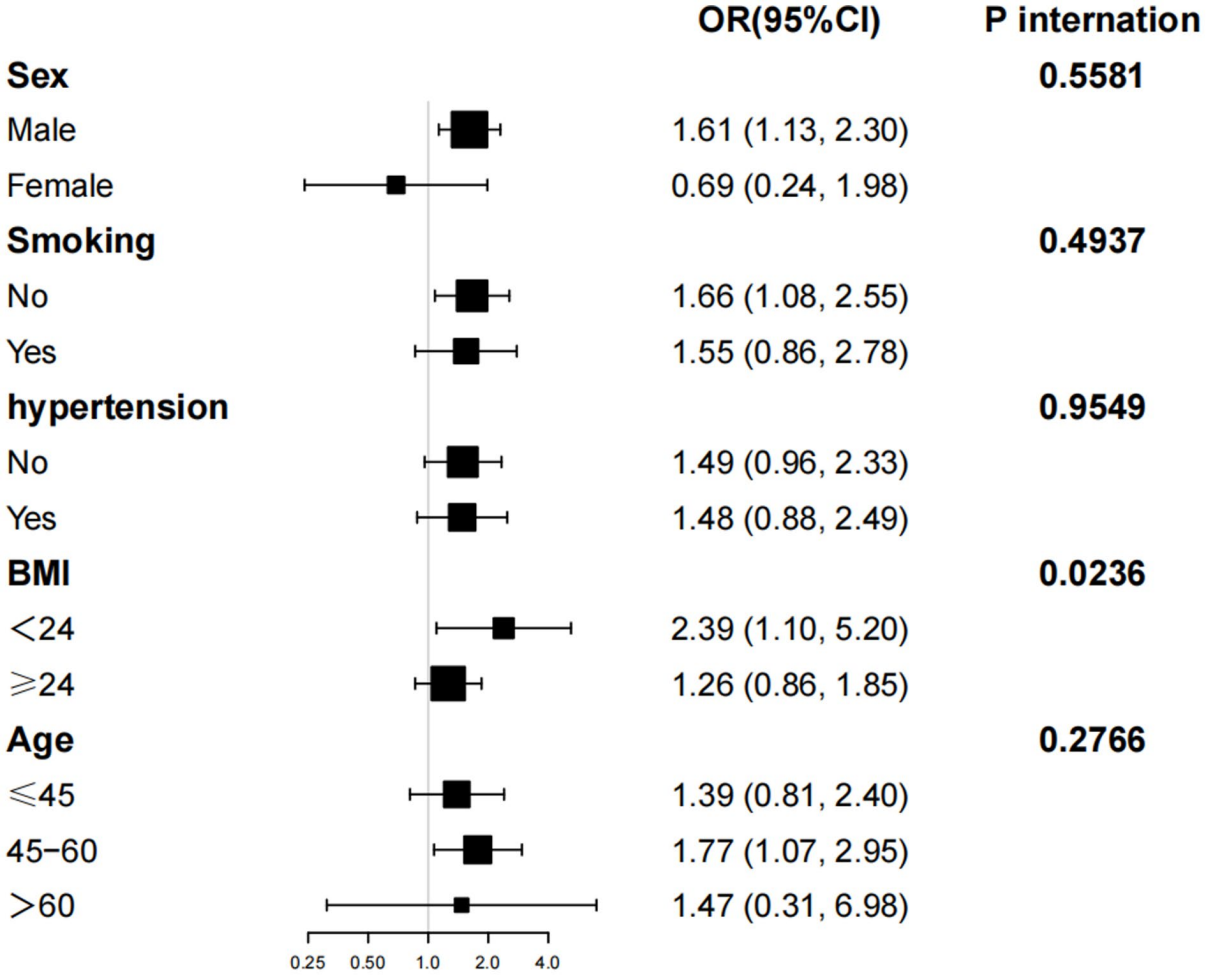
Subgroup analysis for the association of NHHR with EOT2D combined with NAFLD.

Further subgroup analyses and interaction tests, stratified by age, gender, smoking status, hypertension, and BMI, were conducted to assess the robustness of the association between NHHR levels and the risk of NAFLD combined with EOT2D across different subpopulations. Significant associations were observed among participants with a BMI < 24 kg/m^2^ (OR: 2.39; 95% CI: 1.10–5.23). Interaction analyses revealed no significant effect modification by gender, age, BMI smoking status, or hypertension on the positive association between NHHR and NAFLD with EOT2D (*P*-interaction>0.01 for all) (see Figure 3).

### ROC analysis of NHHR for NAFLD comorbid with EOT2D

**Figure 4:**
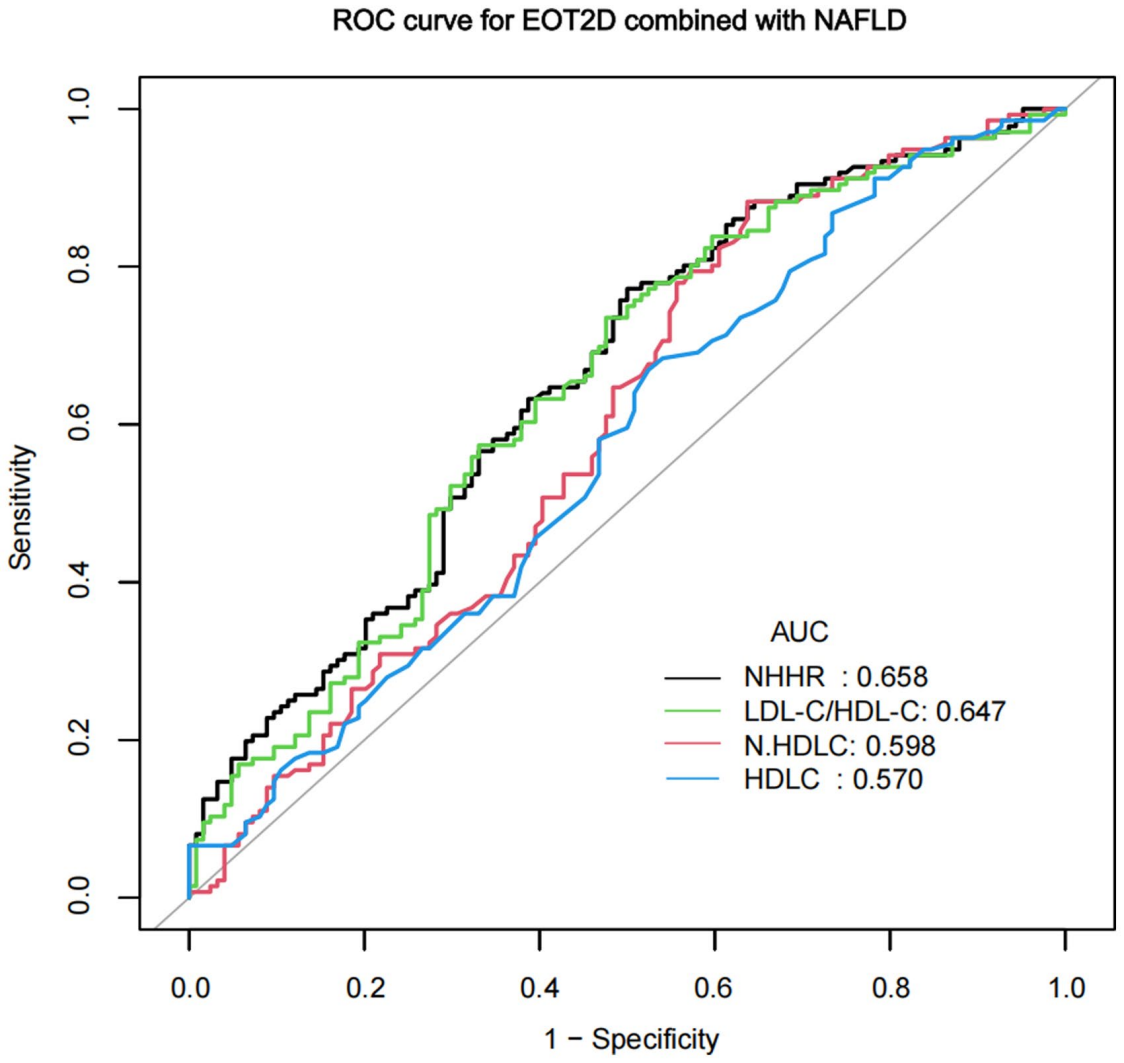
ROC curve of NHHR predicting NAFLD in patients with EOT2D.

In previous studies, HDL-C, non-HDL-C, and LDL-C/HDL-C ratio have been identified as valid predictors of T2DM combined with NAFLD (15, 27). EOT2D combined NAFLD is one type of T2DM combined NAFLD. In this study, we compared NHHR with these factors using ROC curve analysis (Figure 4). ROC curve analysis showed that NHHR had a better diagnostic value with an area under the curve (AUC) greater than that of the other three factors, with an AUC value of 0.658 (95% CI 0.5919–0.7243). The AUC remains at a moderate level, indicating its moderate discriminatory ability for EOT2D with NAFLD. By calculating Jorden’s index, the optimal critical value of NHHR was 3.105. The sensitivity of NHHR for diagnosing EOT2D combined with NAFLD was 77.21%, and the specificity was 50.00%.

## Discussion

Previous studies have identified NHHR as a risk factor for T2DM as well as NAFLD (12, 24). Owing to shared pathogenic mechanisms, non-alcoholic fatty liver disease (NAFLD) and type 2 diabetes mellitus (T2DM) are closely interrelated. The coexistence of these two conditions increases the risk of adverse liver-related outcomes and imposes a heavier burden of extrahepatic complications, making it a significant public health concern. Currently, there is no research investigating the relationship between the non-high-density lipoprotein cholesterol to high-density lipoprotein cholesterol ratio (NHHR) and NAFLD comorbid with EOT2D. The aim of this study was to investigate the correlation between NHHR and EOT2D combined with NAFLD.

The global burden of diabetes is substantial and continues to grow. Early-onset type 2 diabetes (EOT2D), defined by diagnosis before 40 years of age, has witnessed a concerning rise in prevalence over recent decades. This trend parallels the increasing prevalence of obesity within younger populations. EOT2D follows a more aggressive clinical course than later-onset disease, characterized by heightened insulin resistance and accelerated deterioration of beta-cell function. This pathophysiology leads to a more rapid progression of hyperglycemia, consequently conferring a significantly higher risk of diabetes-related complications and premature mortality (28). In addition, the results of a cohort study showed that EOT2D was associated with an increased risk of early-onset overall cancer, as well as a significant association with an elevated risk of diabetes-related cancers and obesity-related cancers (29). Wang et al. (30) showed that in the normal population, the incidence of NAFLD was higher in the lower age group than in other age groups, which reflects the trend of younger NAFLD onset. Alarmingly, people with NAFLD are becoming younger and younger, and young people may experience long-term and early exposure to the diabetic environment. The findings of He et al. (31) found a stronger association between cardiometabolic risk factors and early-onset NAFLD, with early-onset NAFLD also leading to worse outcomes. NAFLD patients under 45 years of age have a significantly higher risk of developing cancer compared to other age groups (32). Epidemiologic data consistently show that patients with T2DM have twice the prevalence of NAFLD (including advanced hepatic fibrosis) and a higher risk of liver-related morbidity (advanced hepatic fibrosis, hepatocellular carcinoma) and mortality than the general population (33). In previous studies, the majority of data on EOT2D have come from children and adolescents (<19 years of age) (34, 35) rather than individuals aged 19–39 years, and adults with early-onset T2DM are severely underrepresented in clinical studies (36). This group of patients has more confidence in their health, which can lead to suboptimal glycemic control and delayed detection of diabetes-related complications and comorbidities. Chen et al. (37) showed that after 1 year of standardized management of T2DM patients, EOT2D patients showed more significant improvements in metabolic markers and greater improvements in FCP, HBA1c, and BMI compared to LOT2D patients. So it is important to pay attention to this specific population as well as to the comprehensive management of their comorbidities and complications.

NHHR is a novel lipid marker newly proposed in recent years, containing all the information related to anti-atherogenic and pro-atherogenic lipid particles, reflecting the balance between lipoproteins and emerging as a potential marker of metabolic disorders (12, 27, 38). There is growing evidence that NHHR can predict various lipid metabolism-related diseases, such as metabolic syndrome and insulin resistance, with a higher diagnostic value than traditional lipid parameters (12, 38, 39). The pathophysiological relationship between T2DM and NAFLD is characterized by insulin resistance, dysregulated hepatic lipid metabolism resulting in steatosis, and hyperinsulinemia-induced metabolic dysfunction. This triad drives a self-perpetuating cycle of glucolipotoxicity, accelerating progression to advanced liver disease and diabetes complications (40). Results of a study suggest that NHHR may be a predictor of screening for NAFLD in the T2DM population (41). Understanding this metric may help identify those who may benefit from early intervention and assist in the next step of clinical decision-making (42).

In this study, 1,158 patients with T2DM were divided into EOT2D and LOT2D groups. Compared with the patients in the LOT2D group, the EOT2D group had a higher proportion of males, a higher BMI, poorer glycemic control, higher NHHR. This is consistent with the findings of Zeitler et al. (28) that patients with EOT2D had higher blood glucose levels and worse overall metabolic profiles at baseline compared to patients with LOT2D. A cross-sectional study in China also showed a higher proportion of men among newly diagnosed EOT2D patients (43). Subsequently, logistic regression modeling confirmed that NHHR is an independent risk factor for EOT2D. We further categorized this group of EOT2D into a combined NAFLD group and an uncomplicated NAFLD group, and NHHR was also significantly higher in the combined NAFLD group than in the uncomplicated NAFLD group. BMI, FINS, FCP, FBG, HbA1c, ALT, AST, ALB, GGT, UA, TG, TC, WBC, N, L, M, and PLT levels were significantly higher in the combined NAFLD group compared with the group without NAFLD. In a study by Cheng et al. (44) it was shown that the MAFLD group exhibited higher levels of biochemical markers such as BMI, SBP, DBP, and TG, and lower levels of HDL-C, which is also in agreement with the results of our study. We compared the basic characteristics of all participants in the four groups grouped by NHHR quartile and showed that participants in the highest NHHR quartile were more likely to be male, younger, had a higher BMI, and were more likely to be smokers and be comorbidly hypertensive, and that participants in this group had higher levels of glucose and HBA1c, and poorer overall metabolic health. Among EOT2D participants, baseline information was analyzed after grouping by NHHR quartiles, which showed higher BMI, increased prevalence of NAFLD, and poorer glycemic and HBA1c control in the higher quartile group. T2DM hyperglycemia leads to alterations in lipid metabolism, including enhanced HDL clearance, reduced apoA-1 transcription, and accelerated HDL glycation (45). Non-HDL-C has become a more reliable predictor of coronary artery disease risk than traditional lipid parameters (46). NHHR, a composite lipid metabolism index that incorporates non-HDL-C, also has better efficacy in predicting T2DM as well as NAFLD (47, 48).

After logistic regression analysis, NHHR proved to be a significant risk factor for EOT2D patients as well as EOT2D patients with combined NAFLD. Gender-stratified analysis revealed that in the male subgroup, the association between NHHR and EOT2D, as well as the composite endpoint of EOT2D and non-alcoholic fatty liver disease (NAFLD), was significantly stronger. In contrast, this association was not statistically significant in the female subgroup. This may be because gender potentially influences the association between NHHR and metabolic outcomes through multiple pathways, such as estrogen promoting high-density lipoprotein synthesis, and men being more prone to visceral fat accumulation and atherosclerotic lipid profiles (49). Visceral fat storage influences insulin metabolism by releasing free fatty acids into the portal venous circulation. This may diminish the liver’s insulin clearance capacity, leading to insulin resistance and hyperinsulinemia (50). The results of the fitted curve analysis showed that NHHR remained a significant risk factor for EOT2D as well as for EOT2D combined with NAFLD after adequate adjustment for confounders. ROC curve analysis showed that NHHR had a greater predictive value for EOT2D combined NAFLD compared to traditional lipid-related metrics such as HDL-C, non-HDL-C, and LDL-C/HDL-C ratio, its moderate AUC suggests it may be most useful as part of a composite risk assessment rather than as a standalone diagnostic tool. NHHR calculation is based on routine lipid panel test reports, requires no additional costs, and demonstrates high feasibility in primary care settings and large-scale population screening. In addition, the correlation between NHHR and the risk of NAFLD was more significant in EOT2D participants with a BMI < 24. EOT2D patients with a BMI < 24 may exhibit “hidden obesity” despite having overall body weight within the normal range—characterized by a predominant accumulation of visceral adipose tissue (VAT) (51). VAT reduces the uptake of free fatty acids (FFAs) while increasing their release. When free fatty acid concentrations in the blood become excessively high, these molecules trigger insulin resistance. Additionally, portal vein blood contains high levels of free fatty acids and cytokines secreted by VAT, which are believed to drive the progression of NAFLD (52). Although their BMI falls within the normal range, lean NAFLD patients often present with sarcopenia. Sarcopenia is now recognized as a progressive pathological process associated with type 2 diabetes mellitus (T2DM), metabolic syndrome, liver disease, and cardiovascular disease (53). It should be clarified that the results of this subgroup analysis are currently preliminary findings and require validation through larger prospective cohort studies. Our findings further underscore the critical importance of targeted health education initiatives and early, sustained clinical interventions in improving metabolic outcomes among young populations.

This hospital-based cross-sectional study ensured cohort consistency and demographic stability. The NHHR measurement demonstrates significant clinical utility due to its cost-effectiveness and accessibility, facilitating early identification of NAFLD risk in EOT2D patients—crucial for guiding targeted interventions. Previous studies have primarily focused on the association between NHHR and populations with T2DM or NAFLD (12, 20, 24), while analyses targeting the EOT2D with NAFLD subgroup have not yet been reported. This is the first study to investigate the association value of NHHR in a specific subgroup of EOT2D patients with NAFLD comorbidity.

Several limitations warrant acknowledgment: the diagnosis of NAFLD relies on ultrasound examination, which can only assess the degree of hepatic steatosis and cannot distinguish between nonalcoholic steatohepatitis (NASH) and fibrosis; this study did not adjust for the use of medications such as lipid-lowering or hypoglycemic agents, thereby potentially introducing bias to the outcomes. Future studies should incorporate detailed medication use data to further validate; Furthermore, due to the cross-sectional nature of this study, we cannot establish a causal relationship between NHHR and EOT2D or EOT2D combined with NAFLD, and the directionality of the association remains uncertain. We will address these through expanded cohorts and prospective studies. These findings hold particular relevance for China, where delayed EOT2D diagnosis affects >50% of diabetes patients due to untimely care access.

## Conclusion

In conclusion, our study suggests that NHHR is an important risk factor for EOT2D patients, especially for EOT2D patients with comorbid NAFLD. Therefore, it is particularly important for us to promptly identify young-onset T2DM patients with comorbid NAFLD in our future clinical work.

## Data Availability

The original contributions presented in the study are included in the article/Supplementary material, further inquiries can be directed to the corresponding authors.

## Glossary

NHHR: non-high-density lipoprotein cholesterol to high-density lipoprotein cholesterol ratio
BMI: body mass index
FINS: fasting insulin
FCP: fasting C-peptide
FBG: fasting blood glucose
HbA1c: Hemoglobin A1c
ALT: alanine transaminase
AST: aspartate transaminase
ALB: albumin
GGT: gamma-glutamyl transferase
BUN: blood urea nitrogen
Cr: serum creatinine
UA: serum uric acid
TG: triglyceride
TC: total cholesterol
HDL-C: high density lipoprotein cholesterol
LDL-C: low density lipoprotein cholesterol
WBC: white Blood Cell Differential
N: neutrophil count
L: lymphocyte count
M: monocyte count
PLT: platelet count
UACR: urine albumin-to-creatinine ratio
ROC: receiver operating characteristic
AUC: areas under the curve
MASLD: metabolic dysfunction-associated steatotic liver disease
T2DM: type 2 diabetes mellitus
EOT2D: early onset type 2 diabetes
LOT2D: late onset type 2 diabetes
