# Supplementary material for: The non-high-density lipoprotein cholesterol to high-density lipoprotein cholesterol ratio is associated with early onset type 2 diabetes with NAFLD: a cross-sectional study in China
Source: Front Nutr. 2025 Oct 31;12:1662125. doi: 10.3389/fnut.2025.1662125 (PMC12616743; doi:10.3389/fnut.2025.1662125)
Supplement: Supplementary file 1 [file Table_1.DOCX]

Supplementary Material

**Supplementary Table S1** Baseline Characteristics of Subjects Based on NHHR Quartile

| **Variable** | **Q1** | **Q2** | **Q3** | **Q4** | ***P*-value** |
| --- | --- | --- | --- | --- | --- |
|  | **N=290** | **N=289** | **N=289** | **N=290** |  |
| Age(years) | 63.89 ± 9.51 | 60.38 ± 10.91 | 58.15 ± 10.95 | 54.74 ± 11.66 | <0.001 |
| Sex(%) |  |  |  |  | <0.001 |
| Male | 162(55.86) | 151(52.25) | 182(62.98) | 206(71.03) |  |
| Female | 128(44.14) | 138(47.75) | 107(37.02) | 84(28.97) |  |
| Smoking(%) |  |  |  |  | 0.006 |
| Yes | 61(21.03) | 46(15.92) | 67(23.18) | 81(27.93) |  |
| No | 229(78.97) | 243(84.08) | 222(76.82) | 209(72.07) |  |
| Hypertension(%) |  |  |  |  | 0.017 |
| Yes | 179(61.72) | 171(59.17) | 156(53.98) | 144(49.66) |  |
| No | 111(38.28) | 118(40.83) | 133(46.02) | 146(50.34) |  |
| EOT2D(%) |  |  |  |  | <0.001 |
| Yes | 40(13.79) | 55(19.03) | 67(23.18) | 98(33.79) |  |
| No | 250(86.21) | 234(80.97) | 222(76.82) | 192(66.21) |  |
| NAFLD(%) |  |  |  |  | <0.001 |
| Yes | 103(35.52) | 143(49.48) | 177(61.25) | 186(64.14) |  |
| No | 187(64.48) | 146(50.52) | 112(38.75) | 104(35.86) |  |
| Diabetic kidney disease(%) |  |  |  |  | 0.699 |
| Yes | 79(27.24) | 82(28.37) | 73(25.26) | 71(24.48) |  |
| No | 211(72.76) | 207(71.63) | 216(74.74) | 219(75.52) |  |
| Disease duration(months) | 120.00 (72.00-192.00) | 120.00 (48.00-169.00) | 96.00 (36.00-168.00) | 72.00 (24.00-144.00) | <0.001 |
| BMI(kg/m^2^) | 24.22 ± 3.54 | 24.94 ± 3.35 | 25.27 ± 3.30 | 25.76 ± 3.42 | <0.001 |
| FINS(uIU/mL) | 6.67 (4.41-10.93) | 7.57 (5.33-11.52) | 8.34 (5.76-12.93) | 10.72 (6.84-16.37) | <0.001 |
| FCP(μg/L) | 1.62 (1.09-2.36) | 1.82 (1.25-2.72) | 2.11 (1.44-2.82) | 2.44 (1.76-3.53) | <0.001 |
| FBG(mmol/L) | 7.22 ± 2.42 | 7.73 ± 2.80 | 7.99 ± 2.90 | 8.60 ± 3.09 | <0.001 |
| HbA1c(%) | 7.83 ± 1.68 | 7.96 ± 1.69 | 8.09 ± 1.80 | 8.54 ± 1.92 | <0.001 |
| ALT(U/L) | 16.30 (13.00-21.60) | 17.70 (13.40-25.10) | 18.50 (13.50-25.90) | 20.80 (14.00-28.40) | <0.001 |
| AST(U/L) | 20.50 ± 7.17 | 20.31 ± 6.69 | 20.60 ± 7.81 | 21.11 ± 8.74 | 0.630 |
| ALB(g/L) | 40.11 ± 3.93 | 40.25 ± 3.81 | 40.09 ± 3.20 | 40.68 ± 3.38 | 0.027 |
| GGT(U/L) | 18.00 (14.00-25.00) | 21.00 (15.00-33.00) | 22.00 (17.00-34.00) | 26.00 (19.00-41.00) | <0.001 |
| BUN(mmol/L) | 6.15 ± 1.69 | 5.83 ± 1.68 | 5.79 ± 1.61 | 5.79 ± 1.53 | 0.018 |
| Cr(μmol/L) | 67.11 ± 17.33 | 64.19 ± 18.07 | 66.26 ± 16.92 | 67.02 ± 19.01 | 0.170 |
| UA(μmol/L) | 302.47 ± 81.00 | 305.80 ± 81.44 | 331.57 ± 83.61 | 353.60 ± 91.82 | <0.001 |
| TG(mmol/L) | 1.07 (0.81-1.44) | 1.43 (1.08-1.96) | 1.70 (1.32-2.47) | 2.54 (1.77-4.16) | <0.001 |
| TC(mmol/L) | 3.91 ± 1.08 | 4.61 ± 0.88 | 5.01 ± 0.93 | 5.50 ± 1.05 | <0.001 |
| HDLC(mmol/L) | 1.33 ± 0.35 | 1.21 ± 0.23 | 1.14 ± 0.21 | 1.02 ± 0.19 | <0.001 |
| LDLC(mmol/L) | 2.29 ± 0.70 | 2.90 ± 0.57 | 3.23 ± 0.63 | 3.49 ± 0.69 | <0.001 |
| WBC(10^9^/L) | 5.85 ± 1.38 | 5.92 ± 1.46 | 6.16 ± 1.42 | 6.56 ± 1.57 | <0.001 |
| N(10^9^/L) | 3.33 ± 1.09 | 3.33 ± 1.07 | 3.48 ± 1.09 | 3.68 ± 1.14 | <0.001 |
| L(10^9^/L) | 1.85 ± 0.54 | 1.95 ± 0.60 | 2.04 ± 0.65 | 2.18 ± 0.66 | <0.001 |
| M(10^9^/L) | 0.46 ± 0.15 | 0.45 ± 0.15 | 0.46 ± 0.14 | 0.48 ± 0.14 | 0.029 |
| PLT(10^9^/L) | 188.13 ± 45.83 | 195.51 ± 48.06 | 197.20 ± 49.44 | 208.61 ± 52.00 | <0.001 |
| UACR(mg/g) | 10.27 (5.22-35.27) | 12.68 (6.37-35.76) | 10.14 (5.49-30.52) | 11.51 (5.77-28.52) | 0.246 |

Abbreviations: BMI, body mass index; FINS, fasting insulin; FCP, fasting C-peptide; FBG, fasting blood glucose; HbA1c, Hemoglobin A1c; ALT, alanine transaminase; AST, aspartate transaminase; ALB, albumin; GGT, gamma-glutamyl transferase; BUN, b**lood urea nitrogen; Cr, serum creatinine; UA, serum uric acid;** TG, triglyceride; TC, total cholesterol; HDL-C, high density lipoprotein cholesterol; LDL-C, low density lipoprotein cholesterol; NHHR, non-high-density lipoprotein cholesterol to high-density lipoprotein cholesterol ratio; WBC, white Blood Cell Differential; N, neutrophil count; L, lymphocyte count; M, monocyte count; PLT, platelet count; UACR, urine albumin-to-creatinine ratio.

**Supplementary Table S2** Baseline characteristics of early-onset diabetes based on NHHR quartile

| **Variable** | **Q1**  **N=40** | **Q2**  **N=55** | **Q3**  **N=67** | **Q4**  **N=98** | ***P*-value** |
| --- | --- | --- | --- | --- | --- |
| Age(years) | 54.17±10.19 | 48.75±12.82 | 48.15±11.01 | 44.66 ±9.79 | <0.001 |
| Sex(%) |  |  |  |  | 0.207 |
| Male | 32 (80.00) | 35 (63.64) | 48 (71.64) | 76 (77.55) |  |
| Female | 8 (20.00) | 20 (36.36) | 19 (28.36) | 22 (22.45) |  |
| Smoking(%) |  |  |  |  | 0.846 |
| Yes | 11 (27.50) | 16 (29.09) | 22 (32.84) | 26 (26.53) |  |
| No | 29 (72.50) | 39 (70.91) | 45 (67.16) | 72 (73.47) |  |
| Hypertension(%) |  |  |  |  | 0.438 |
| Yes | 22 (55.00) | 26 (47.27) | 27 (40.30) | 41 (41.84) |  |
| No | 18 (45.00) | 29 (52.73) | 40 (59.70) | 57 (58.16) |  |
| NAFLD(%) |  |  |  |  | <0.001 |
| Yes | 10 (25.00) | 23 (41.82) | 41 (61.19) | 62 (63.27) |  |
| No | 30 (75.00) | 32 (58.18) | 26 (38.81) | 36 (36.73) |  |
| Diabetic kidney disease(%) |  |  |  |  | 0.109 |
| Yes | 12 (30.00) | 24 (43.64) | 21 (31.34) | 24 (24.49) |  |
| No | 28 (70.00) | 31 (56.36) | 46 (68.66) | 74 (75.51) |  |
| Disease duration(months) | 162.00 (96.00-240.00) | 120.00 (36.00-204.00) | 120.00 (54.00-210.00) | 102.00 (36.00-189.00) | 0.115 |
| BMI(kg/m^2^) | 24.62±2.13 | 25.55±3.29 | 25.81±3.71 | 26.34±3.70 | 0.063 |
| FINS(uIU/mL) | 5.70 (4.13-8.00) | 7.06 (4.46-11.33) | 9.90 (5.82-15.44) | 10.47 (6.75-16.94) | <0.001 |
| FCP(μg/L) | 1.36 (0.81-1.87) | 1.78 (1.09-2.52) | 1.96 (1.25-2.70) | 2.17 (1.63-3.32) | <0.001 |
| FBG(mmol/L) | 6.42±1.69 | 7.93±2.78 | 7.90±2.42 | 9.28±3.34 | <0.001 |
| HbA1c(%) | 7.56±1.42 | 8.20±1.87 | 8.27±1.63 | 8.80±1.75 | 0.001 |
| ALT(U/L) | 17.65 (14.62-22.05) | 17.70 (13.30-25.85) | 18.90 (13.80-31.30) | 22.15 (14.90-32.60) | 0.058 |
| AST(U/L) | 19.45±6.27 | 19.80±7.45 | 20.00±7.95 | 20.85±8.48 | 0.747 |
| ALB(g/L) | 40.27±3.96 | 40.18±3.46 | 40.62±3.30 | 41.73±3.81 | 0.035 |
| GGT(U/L) | 21.50 (15.00-26.00) | 26.00 (14.50-37.00) | 24.00 (16.00-33.00) | 28.50 (18.00-45.75) | 0.014 |
| BUN(mmol/L) | 5.81±1.40 | 5.88±1.57 | 5.68±1.56 | 5.81±1.57 | 0.907 |
| Cr(μmol/L) | 68.80±16.24 | 66.95±18.62 | 66.30±18.44 | 64.02±17.27 | 0.495 |
| UA(μmol/L) | 321.57±67.03 | 309.80±78.77 | 338.01±86.52 | 351.67±77.90 | 0.012 |
| TG(mmol/L) | 1.13 (0.90-1.38) | 1.45 (1.13-1.71) | 1.66 (1.35-2.48) | 2.59 (1.83-4.40) | <0.001 |
| TC(mmol/L) | 3.76±1.13 | 4.40±0.86 | 5.06±0.90 | 5.35±1.01 | <0.001 |
| HDLC(mmol/L) | 1.28±0.34 | 1.16±0.22 | 1.15±0.20 | 0.99±0.19 | <0.001 |
| LDLC(mmol/L) | 2.28±0.80 | 2.75±0.55 | 3.27±0.61 | 3.41±0.69 | <0.001 |
| WBC(10^9^/L) | 5.90±1.60 | 6.03±1.41 | 6.31±1.53 | 6.55±1.48 | 0.041 |
| N(10^9^/L) | 3.39±1.20 | 3.41±0.94 | 3.64±1.21 | 3.61±1.01 | 0.467 |
| L(10^9^/L) | 1.83±0.54 | 1.96±0.63 | 2.02±0.64 | 2.25±0.65 | 0.001 |
| M(10^9^/L) | 0.46±0.19 | 0.47±0.15 | 0.45±0.15 | 0.48±0.14 | 0.621 |
| PLT(10^9^/L) | 202.40±51.14 | 207.29±49.00 | 201.43±53.38 | 213.95±52.61 | 0.422 |
| UACR(mg/g) | 10.39 (4.54-34.03) | 20.82 (6.97-78.91) | 10.30 (5.60-43.16) | 10.30 (4.92-27.84) | 0.141 |

Abbreviations: BMI, body mass index; FINS, fasting insulin; FCP, fasting C-peptide; FBG, fasting blood glucose; HbA1c, Hemoglobin A1c; ALT, alanine transaminase; AST, aspartate transaminase; ALB, albumin; GGT, gamma-glutamyl transferase; BUN, b**lood urea nitrogen; Cr, serum creatinine; UA, serum uric acid;** TG, triglyceride; TC, total cholesterol; HDL-C, high density lipoprotein cholesterol; LDL-C, low density lipoprotein cholesterol; NHHR, non-high-density lipoprotein cholesterol to high-density lipoprotein cholesterol ratio; WBC, white Blood Cell Differential; N, neutrophil count; L, lymphocyte count; M, monocyte count; PLT, platelet count; UACR, urine albumin-to-creatinine ratio.

**Supplementary Table S3(1)** Relationship between NHHR and EOT2D in the male subgroup

|  | **Model 1** |  |  | **Model 2** |  |  | **Model 3** |  |
| --- | --- | --- | --- | --- | --- | --- | --- | --- |
|  | **OR(95%CI)** | ***P*-value** |  | **OR(95%CI)** | ***P*-value** |  | **OR(95%CI)** | ***P*-value** |
| NHHR | 1.32 (1.13, 1.53) | 0.0003 |  | 1.30 (1.11, 1.53) | 0.0013 |  | 1.21 (1.02, 1.44) | 0.0304 |
| Q1 | Ref |  |  | Ref |  |  | Ref |  |
| Q2 | 1.23 (0.71, 2.11) | 0.4607 |  | 1.22 (0.70, 2.15) | 0.4842 |  | 1.15 (0.64, 2.05) | 0.6362 |
| Q3 | 1.46 (0.88, 2.42) | 0.1480 |  | 1.40 (0.82, 2.38) | 0.2172 |  | 1.20 (0.69, 2.09) | 0.5269 |
| Q4 | 2.37 (1.47, 3.84) | 0.0004 |  | 2.49 (1.49, 4.16) | 0.0005 |  | 1.94 (1.12, 3.35) | 0.0178 |
| *P* for trend |  | 0.0002 |  |  | 0.0003 |  |  | 0.0156 |

Model 1: Non-adjusted

Model 2: Adjusted for Diabetes duration,smoking,BMI,hypertension

Model 3: Adjusted for Diabetes duration,smoking,BMI,hypertension,HbA1c,ALT,AST,UA,WBC,PLT,UACR

Abbreviations: CI, confidence interval;Ref,Reference

**Supplementary Table S3(2)** Association between NHHR and EOT2D combined with NAFLD in the male subgroup

|  | **Model 1** |  |  | **Model 2** |  |  | **Model 3** |  |
| --- | --- | --- | --- | --- | --- | --- | --- | --- |
|  | **OR(95%CI)** | ***P*-value** |  | **OR(95%CI)** | ***P*-value** |  | **OR(95%CI)** | ***P*-value** |
| NHHR | 1.88 (1.38, 2.58) | <0.0001 |  | 1.82 (1.31, 2.53) | 0.0004 |  | 1.55 (1.08, 2.23) | 0.0166 |
| Q1 | Ref |  |  | Ref |  |  | Ref |  |
| Q2 | 2.00 (0.70, 5.70) | 0.1947 |  | 1.85 (0.62, 5.51) | 0.2695 |  | 2.05 (0.65, 6.49) | 0.2204 |
| Q3 | 6.00 (2.21, 16.31) | 0.0004 |  | 5.73 (2.01, 16.34) | 0.0011 |  | 5.91 (1.84, 18.98) | 0.0028 |
| Q4 | 5.14 (2.04, 12.98) | 0.0005 |  | 4.70 (1.76, 12.51) | 0.0020 |  | 3.58 (1.24, 10.38) | 0.0187 |
| *P* for trend |  | 0.0001 |  |  | 0.0006 |  |  | 0.0146 |

Model 1: Non-adjusted

Model 2: Adjusted for Diabetes duration,smoking,BMI,hypertension

Model 3: Adjusted for Diabetes duration,smoking,BMI,hypertension,HbA1c,ALT,AST,UA,WBC,PLT,UACR

Abbreviations: CI, confidence interval;Ref,Reference

**Supplementary Table S4(1)** Relationship between NHHR and EOT2D in the female subgroup

|  | **Model 1** |  |  | **Model 2** |  |  | **Model 3** |  |
| --- | --- | --- | --- | --- | --- | --- | --- | --- |
|  | **OR(95%CI)** | ***P*-value** |  | **OR(95%CI)** | ***P*-value** |  | **OR(95%CI)** | ***P*-value** |
| NHHR | 1.41 (1.09, 1.82) | 0.0087 |  | 1.33 (1.02, 1.73) | 0.0356 |  | 1.32 (0.97, 1.79) | 0.0791 |
| Q1 | Ref |  |  | Ref |  |  | Ref |  |
| Q2 | 2.54 (1.08, 6.00) | 0.0331 |  | 2.55 (1.06, 6.18) | 0.0374 |  | 2.31 (0.93, 5.70) | 0.0697 |
| Q3 | 3.24 (1.36, 7.74) | 0.0082 |  | 3.31 (1.35, 8.31) | 0.0089 |  | 2.74 (1.08, 6.97) | 0.0345 |
| Q4 | 5.32 (2.24, 12.65) | 0.0002 |  | 4.85 (2.00, 11.79) | 0.0005 |  | 3.90 (1.51, 10.09) | 0.0050 |
| *P* for trend |  | <0.0001 |  |  | 0.0003 |  |  | 0.0055 |

Model 1: Non-adjusted

Model 2: Adjusted for Diabetes duration,smoking,BMI,hypertension

Model 3: Adjusted for Diabetes duration,smoking,BMI,hypertension,HbA1c,ALT,AST,UA,WBC,PLT,UACR

Abbreviations: CI, confidence interval;Ref,Reference

**Supplementary Table S4(2)** Association between NHHR and EOT2D combined with NAFLD in the female subgroup

|  | **Model 1** |  |  | **Model 2** |  |  | **Model 3** |  |
| --- | --- | --- | --- | --- | --- | --- | --- | --- |
|  | **OR(95%CI)** | ***P*-value** |  | **OR(95%CI)** | ***P*-value** |  | **OR(95%CI)** | ***P*-value** |
| NHHR | 2.00 (0.98, 4.10) | 0.0586 |  | 1.79 (0.83, 3.85) | 0.1348 |  | 0.67 (0.22, 2.03) | 0.4819 |
| Q1 | Ref |  |  | Ref |  |  | Ref |  |
| Q2 | 2.45 (0.40, 15.25) | 0.3353 |  | 1.68 (0.24, 11.74) | 0.6019 |  | 0.35 (0.03, 3.69) | 0.3805 |
| Q3 | 2.70 (0.43, 16.94) | 0.2891 |  | 1.88 (0.26, 13.41) | 0.5273 |  | 0.52 (0.05, 5.65) | 0.5903 |
| Q4 | 5.25 (0.85, 32.43) | 0.0743 |  | 3.57 (0.51, 24.93) | 0.1987 |  | 0.27 (0.02, 3.69) | 0.3279 |
| *P* for trend |  | 0.0654 |  |  | 0.1485 |  |  | 0.5143 |

Model 1: Non-adjusted

Model 2: Adjusted for Diabetes duration,smoking,BMI,hypertension

Model 3: Adjusted for Diabetes duration,smoking,BMI,hypertension,HbA1c,ALT,AST,UA,WBC,PLT,UACR

Abbreviations: CI, confidence interval;Ref,Reference
